# Abstracts and Gleanings

**Published:** 1878-02-20

**Authors:** 


### *ABSTRACTS AND GLEANINGS.*

### GRAVES' DISEASE.

The University Dispensary has been particularly rich in cases of Graves' disease. Upon one occasion, last spring, I was able to bring five thoroughly marked cases of this affection before you, three of which occurred in boys. The patient to-day is a sewing girl, 20 years old, and a resident of this city. Some years ago, she had a severe attack of erysipelas; she has suffered, as long as she can remember, from palpitation of the heart, and has always been easily fatigued. Her monthlies have lately been irregular; in fact, she has not menstruated at all for the past three months. There has been much leucorrhœa and pain in the back. The girl probably has some uterine disease, and is of a highly nervous temperament. Her thyroid gland is enlarged, and there is a very slight thyroid thrill. There is no exophthalmia, however.

Graves' disease occurs most frequently in females of a pronounced nervous temperament. It may occur, however, in the male. The causes which produce this disease are anxiety, overwork, improper food, and insufficient clothing. In females, the predisposing cause is very frequently some uterine disorder.

The prominent symptoms are protrusion of the eyeballs or exophthalmia, enlargement of the thyroid gland, and disturbed cardiac action. To these is often added marked anæmia. The degree to which these symptoms exist varies in different cases. The exophthalmia and goitre may be exceedingly marked, or scarcely perceptible. The disturbance of the heart's action is generally functional and very marked, with an exceedingly rapid pulse, and sometimes a musical, systolic, blowing murmur, due to aæmia.

Let us consider the symptoms separately: First. As regards the enlargement of the thyroid gland; as a rule, both lobes are equally affected. The thyroid gland is highly vascular, and the arteries leading to it are very tortuous. When, then, there is violent arterial over-action, we should be prepared to find pulsation and thrill over the gland. The nature of the thyroid enlargement points strongly to the view that it is due to a dilated and enlarged condition of the vessels.

The exophthalmia is so extreme in some cases that the globes of the eyes can not be covered by the lids, and it becomes necessary to protect them from injury by exposure to air and dust. This exophthalmia seems to be due to the distention of the vessels of the post-ocular tissues, with, perhaps, some hypertrophy of the cellulo-fatty tissues behind the globe. The disturbance of the heart's action is usually the earliest and frequently the most constant of the symptoms. There is very rarely any organic disease at first, though hypertrophy may subsequently supervene. If there is anæmia coexistent, it is not unusual to find anæmic murmurs at the base of the heart.

In lookihg for a common cause for the three symptoms above mentioned, it is probably to be found in a morbid condition, with enfeebled activity of the cervical ganglia of the sympathetic and of the cardiac plexus of nerves. In a few cases actual lesions of the nervous ganglia have been found. There are a few special symptoms occasionally present, which can be explained in the same manner, such as sudden flushing of the face, with violent thr bbing of vessels, and sensations of fulness, or vertigo; local unaliteral sweatings about the head; local modifications in nutrition, such as irregularities in the growth of hair, etc.

The diagnosis of Graves' disease can present but few difficulties if attention be paid to the characteristic features above noted. It is really a very curable affection in many instances, provided it come under treatment at an early stage, and the hygienic conditions can be rendered favorable. Even when cure can not be effected, the troublesome symptoms can be held in check.

In the treatment, care must be had in the removal of the causes, and in securing rest, good food, change of scene, and entire release from anxieties. The various functions must be attended to, and any local disorder in females removed by suitable treatment. The best remedies are iron, digitalis, ergot, and bromide of potassium. Digitalis is the most valuable remedy for controlling the functional disturbance of the heart. It may be given in doses of from ten to fifteen drops, three to four times daily. Iron is also very useful. I shall order this patient from ten to thirty minims of dialyzed iron, three to four times daily. The iron may be combined with the digitalis. Ergot may be given internally, with a view to influencing the contractility of the walls of the arteries. I have obtained most excellent results from the injection of diluted ergotina into the substance of the enlarged thyroid gland. The needle may be introduced to the depth of half an inch, and from six to ten minims of a solution, containing ninety-six grains of ergotina to one fluid ounce of distilled water, injected. Bromide of potassium is frequently called for, partly on account of the general nervous condition, but chiefly to assist the digitalis, or ergot, in controlling the irregular action of the heart and arteries.—*Medical and Surgical Reporter*.

[These iodide of iron would seem to be indicated iu this condition—Iodoform and iron, Iron quinine and strychinia—Iodide potass. with com. Tinc. getian, etc.—Ed. Record.]

---

LISTER'S ANTISEPTIC METHOD.

M. Schuppert, M. D., in charge of the New Orleans Charity Hospital, says in the *New Orleans Medical and Surgical Journal:*

"From what I had seen in a few of the larger hospitals, in Germany, I stated in the publication mentioned, that a revolution had taken place in one of the most important departments of surgery, and I prognosticated with the speedy downfall of the old methods of treating wounds, and the general acceptance of Lister's antiseptic method, a new era in surgical practice, although this method was yet in its infancy, and the number of surgeons a small one who were then ready to confess its superiority over all other methods. One of the great obstacles in the way of a more extensive adoption of the teachings of the distinguished Scotchman, lays in the idea, that this treatment was based upon a theory to which many surgeons were then opposed. Yet Lister himself did not insist upon the adoption of any theory in advancing his method. He had, by a succession of trials, finally come to recommend his empirical treatment, which, according to the experience of Prof. Volkmann, in Halle, promised results at that time unheard of in surgical practice, and surpassing even the keenest expectations.

"From what I had seen, and from the statements of that trustworthy prefessional expert—statements corroborated by hundreds of eye-witnesses—it could not longer be a doubtful issue with me to prognosticate the final universal adoption of these revolutionary ideas. What I had predicted to come to pass has thus been fulfilled in extenso.

"Amongst the surgeons in Germany, at least, there is at present but one voice, and that is in favor of Lister's antiseptic treatment of wounds. Slowly it has also broken ground in England and in Italy, and even France begins to be heard of. At the time I visited Paris, in 1875, the name of Lister and his antiseptic treatment were things unkown in the Lariboisiere, a res innota at the once celebrated Hotel Dieu. Shall it be said of this, our country, that it is behind others, and slow in urging the necessity of the general adoption of the antiseptic method? Besides the security which this method renders to limb and life, the almost exceptional good results it promises in treating the most dangerous injuries of joints, the protection it guarantees against accidental wound diseases, pyæmic, septicæmic and diptheritic fevers, the safety it offers against inflammatory conditions so often associated with large wounds, it proffers also the prospect of healing wounds by first intention, be it even wounds of the largest size, as in flap or circular amputations. Next to the security, it exhibits the shortening of the time to a patient's final recovery. Are not these virtues sufficient to make its general adoption an unconditional necessity, and ought not the surgeon to be held morally (if not legally) responsible for every life lost, if he neglects to adopt those principles which guarantee such a successful and safe issue?

"It cannot be denied, that under other and quite different treatment also good results are occasionally obtained, yet they will not stand a proper scrutiny. Such results are accidental, obtained under favorable conditions, which if absent would have had quite a different issue; whereas in Lister's antiseptic treatment, we recognize those virtues which promise the propitious results under all, even the most adverse circumstances. Who would have the hardihood to perform the operation of tracheotomy, for instance, in a room where diptheritic wound diseases were present, or perform any other operation in the presence of hospital gangrene, erysipelas, or other diseases, their dangerous effluvia towards wounds being amply known? Yet under the protecting folds of Lister's antiseptic method, we may even expose our patients without the risk of an infection, if only its directions are strictly adhered to, but to the minutest prescriptions. A great error is occasionally committed in changing one or the other element composing Lister's method. What do we not hear called an antiseptic treatment? Some believe that if they only stuff the wounds with so-called antiseptic materials as jute, or are using carbolic acid (mixed with oil, not knowing that the oil neutralizes to a great extent the antiseptic effects of the phenol); others think that if they cover the wounds with antiseptic gauze, all requirements of an antisepsis have been fulfilled. Even in Germany, some prominent surgeons had thought proper to leave out one or the other element composing Lister's method, as, for instance, the spray, building up a theory of their own about the efficiency or non-efficiency of one or the other material; yet it is known, that not one of them has as yet arrived at the astonishing results of Prof. Volkmann, of Halle, and most of them have returned to the original proper 'Listering.'"

---

TREATMENT OF CHRONIC GONORRHŒA.

Dr. Gschirhakl (*Vierteljahresschrift f. Dermatol. und Syphilis*, 1878, No. 4). Since the endoscope has rendered the urethral lining accessible to inspection, the topical treatment of chronic gonorrhœa has assumed a more rational basis. The remedy can be applied to the very spot we wish to treat; we can dispense with indiscriminate injections, and the uncertainty of bougies smeered over with ointments. The solid caustic need be used but in exceptional cases, because we can employ the remedy in a milder form; we can use it as a solution applied by means of a camel's hair brush. This method has been employed with success by Tarnowsky, Grimfeld, and Fenger; and Dr. G. has also employed it in the past two years. The apparatus necessary for this topical medication, consists of the urethroscope, a metallic catheter with its beak cut off, and a set screw at the other end, an obturator to fit in the catheter, a camel's hair brush set in a long, spiral wire, and a small syringe.

After the exact location of the ulcer, granulations, or swelling of the urethral membrane has been ascertained with the urethroscope, the catheter, with the obdurator securely fastened by the screw, is introducod as far as the diseased spot. While the catheter is held in this position, the obdurator is withdrawn, and the previously moistened camel's hair brush passed in instead. The medicine is then dropped from the syringe into the catheter; it will run down the tube, collect above the brush, and soak into the latter. This done, the catheter must be withdrawn a little in order to get the brush on the diseased surface; and, finally, the brush can be rotated if the extent of the disease demands it. The application done, the brush is drawn back into the catheter, and both are withdrawn from the urethra. The remedies oftenest used are silver nitrate (5 to 10 grs. solution) and aluminated copper (10 grs.—*Chicrgo Medieal Journal.*

WARTS.

In the American Dermatological Association, Dr. White said that his method of treating warts was, after paring them down, to have them moistened, and pulverized muriate of ammonia to be applied, as much as will adhere. In the case of molluscum he had had the remedy bored into the masses with a stick moistened and dipped in the same.

Dr. Hardaway had treated ordinary warts successfully by means of electrolysis; transfixing the base with a needle attached to the negative pole, and placing the positive in the hand of the patient, he passed a current from two or three cells through it, for several successive days.

Dr. Fox had not given much treatment to his cases of molluscum; where they were cut off for microscopic examination he had thrust a stick of nitrate of silver into the base. This he considered the best treatment.

Dr. Duhring had met with molluscum contagiosum several times in private practice, but it was a rare disease among the upper classes in Philadelphia; many of the gentlemen present had seen it only among the lower classes of society.

Dr. Wigglesworth had himself had molluscum contagiosum in half a dozen scattered tubercles, which began to appear about two weeks after squeezing the contents from the tubercles on a case of this disease; he was, however, skeptical in regard to the contagiousness of the disease in question.

ERYSIPELAS TREATED BY SILICATE OF SODA.

Alwarenga reports that he has used this soluble glass in forty-eight cases of erysipelas with good and rapid effect. The cases, which were of all forms of the disease, disappeared, on an average, in four days and twenty-two hours, whereas ordinary cases run a course of eight or nine days. The material is first neutralized with an alkali, if it has an acid reaction, and is then diluted with seven or eight times its weight of water. The parts affected are to be painted with the preparation daily, morning and evening, which is allowed to dry upon the skin. After four or five days, when the fever, redness and swelling have disappeared, the substance is removed by laying cloths wet with emulsion of sweet almonds upon the parts. Applied to the sound skin, the silicate produces the sensation of cold, contraction of its tissues, and lowering of its temperature.—*Il Raccoglitore Med.*, 1877.

URTICARIA AND QUININE.

Dr. J. H. Claiborne, of Petersburg, Virginia, alluding to a report of three cases of eruption attributed to quinia, which were published in the New York *Medical Record*, writes that such instances are not at all uncommon in his section of Virginia—Tide-water Virginia. In a communication to the above named paper, he says: "Such cases have certainly not been unusual in my practice for the last twenty years; and for a long time, misled by the statements and opinions of the patients themselves, I attributed this urticaria myself to the quinine administered. I remarked, however, after treating quite a number of cases, that this urticaria not only always appeared in malarious districts, but generally in old intermittent fever patients, and that it observed a periodicity corresponding with the variety of intermittent to which the patient had been most subject—quotidian, tertian or quartan—and that it supplemented the paroxysm, recurring, in other words, on the day and at the hour when the patient ordinarily had his chill. I at once concluded that the quinine, instead of being the cause, should be the cure of the malady; that the fault, after all, lay, as usual, with the *ingesta*, and that after cleaning up the prima viæ, and preparing the way for the proper absorption of the quinine, I should find the bane an antidote. I invariably, thereafter, commenced the treatment with a moderate dose of calomel, bicarb. of soda, and Dover's powder—the latter being almost always rendered necessary by the state of general discomfort and irritation in which I found the patient; and, after a gentle catharsis, gave quinine in appropriate doses, and at such intervals as the expected return of the paroxysm required. If there were much irritability of stomach, I added a little opium and creosote to the quinine pills, and have yet to see the patient who discovered that he was taking the much-dreaded alkaloid, however much he proclaimed the idiosyncrasy which prevented his taking it.—*Drug. Circular.*

---

NEW METHOD OF REDUCING DISLOCATIONS OF THE SHOULDER.

In the *Gazette Medicale de Paris* (quoted in the *Paris Medicale,* March 9, 1876), Dr. Kuhn, of Elbeuf, describes a new method of reducing dislocations of the shoulder. He calls attention to the fact that there is a loss of force, due to the scapula following the traction made on the humerus in the method ordinarily employed to reduce luxations of the shoulder point. He claims, on the contrary, that by making humerus the fixed point, and reducing the scapula, there is no loss of power, and the resistance of those powerful muscles, the pectoralis major and latissimus dorsi, is obviated. With a passing reference to anæsthetics, and the prejudice which some prrctitioners entertain against their use, he proceeds to the modus operandi. A wedge-shaped cushion is placed in the axilla, the base of the wedge being downward; the surgeon, standing at the patient's side, lightly draws the arm downward, and at the same time presses it firmly against the pad in the axilla, so as to make it into a lever of the first kind; then, taking the interior angle of the scapula in the other hand, he raises that bone and gives it a sesaw motion. Coaptation soon follows, the two parts returning to their natural position by a simultaneous effort made on the lower extremity of the humerus and the inferior angle of the scapula. If the head of the humerus be displaced forward, the angle of the scapula should be directed outward at the same time that it is raised. It should be directed inward if the dislocation be backward. If any difficulty be experienced in making the reduction, the task of holding and directing the arm should be confided to an assistant.—*Ec. Med. and Surg. Jour.*

---

HEMORRHOIDS.

A. J. Howe, M.D., in *Eclectic Medical Journal,* says: Within the past six months I have treated six cases of hemorrhoids by the injecting method. At first I employed carbolized oil, but now I find that carbolized water does just as well. With a hypodermic syringe I have thrown into each prominent pile tnmor a fluid drachm or less of a dilute solution of carbolic acid—say eight parts of water to one of the former. The operative procedure produces a stinging pain for a few minutes, yet the discomfort is not great. A certain amount of soreness has to be endured for three or four days, when the real work of repair begins, and the reparative action continues until the injected tumor vanishes. It will hasten the desired result in some instances, if a second or even third injection be employed, especially if the recuperative action be sluggish. One injection will do in favorable cases.

If the hemorrhoids be of the internal variety, and have to be forced down by straining, as at stool, there will generally be two or three compartments of the extruded mass that need injecting; and the result, though good, is not so satisfactory as is obtained from the injection of external hemorrhoids.

While the cure is going on the anal folds may be rubbed daily with cosmoline or almost any cooling and soothing ungent. Stramonium ointment is an excellent application in most cases. The patient need not necessarily be laid up a day, though a degree of soreness will be experienced for two or three days.

The internal use of small doses of sulphur hastens the curative process, for the agent renders the stools soft and pasty. Either looseness or constipation of the bowels aggravates piles.

VIBURNUM.

Dr. DeCrow, in *Scudder's Journal*, remarks:

Of viburnum I claim it to be a specific to prevent habitual abortion or miscarriage, also, to arrest it at whatever stage before the fourth month and after, if the membranes are not ruptured and the amniotic fluid is not discharged. It has never failed in my practice to carry my patient to full term of gestation who has aborted frequently before, I prescribe R.—Tinct. Viburnun Prun., dr. j: aqua pura, oz. iv. M. Teaspoonful every 5 hours. Continue medicine until after seventh month.

To prevent a threatened miscarriage I use an infusion, oz. j of the green bark off the root carefully dried, or green when I can get it, to Oj of boiling water; stepe for thirty minutes; tablespoonful every 15 to 30 minutes until relieved of uterine pains, then every 30 to 60 minutes for 24 to 36 hours, owing to the condition of my patient. I administer nothing else unless there is severe lumbar pains, then I give R.—Tinct. Macrotys, dra. ss; Water, oz. iv. M. Teaspoonful every hour, keeping my patient in a recumbent position for a day or two.

I say again, it is the specific in abortion or miscarriage if properly prepared and used. Not only in abortion have I found it a valuable remedy, but in the treatment of diseases of the reproductive organs of the female. In dysmenorrhœa with deficient menses, uterine colic, in ladies who at the menstrual period have such severe lumbar and bearing down uterine pains, I have found it a most excellent remedy. In these cases I use the specific tincture, varying my dose from grs. ss to oz. ij., water drs. iv., a teaspoonful every half hour, owing to the severity of my case. I should not like to practice without Viburnum close at hand.

SHALL MEDICAL WITNESSES RECEIVE A PROPER COMPENSTION?

Dr. Thomas J. Dill, of Fort Wayne, Ind., has had a controversy with the conrts of his city, and came off second best. He was called to testify in a case of supposed rape. In regard to matters of fact he testified so far as he knew, but when asked his opinion in regard to certain matters, he refused to testify unless paid such equivalent for the service as he was in the habit of charging in his office. The Judge sent him to jail for contempt of court. He was brought from the jail on a writ of habeas corpus, and the writ was argued before the same Judge, it is said, who of course sustained his former ruling, and remanded the prisoner to the sheriff. Dr. Dill, thinking it better to give his opinion than remain in jail, gave the required testimony and was released from custody. A fellow physician of Dr. Dill's was compelled to testify under similar circumstances. The Allen County Medical Society have taken steps to make this a test case before the supreme court, and have called upon other county societies for pecuniary assistance.—*The American Practitioner*.

ELEPHANTIASIS OF THE PENIS.

Duffy gives the notes of quite a remarkable case. The patient (the age is not reported, nor is it stated whether he was white or black) was apparently in robust health, and had never had any disease of the genitalia. Two weeks before, the penis had become erect, and had so remained, without interval of relaxation, up to the time he came under notice. At that date the organ was of average size, and exhibited no symptoms of disease other than the persistent erection. Although the penis was not painful, either at this time nor at any subsequent period, it was in such a hyperæsthetic condition that all moving had to be avoided. At the end of two weeks the erection was still present, and the organ had increased in size. Half way between the corona glandis and symphysis pubis the circumference measured seven inches. The circumference at this point was greater than elsewhere, the organ having a somewhat spindle form. After this the penis increased rapidly in volume, growing equally in all its parts. The skin soon became so distended and tense, that to avoid a rupture a lateral longitudinal incision was made on either side. In three months the circumference of the organ measured twelve inches, at which time it was amputated by the loop of the galvano-cautery applied closely to the pubis. The stump healed kindly.—*Phil. Med. Times*, 1876.

---

AMERICAN GUM ARABIC.

The mesquite gum of Western Texas is almost identical with gum arabic. During the past year it has become an article of export, some 12,000 pounds having been gathered in Bexar county, and as much more between that and the coast. This gum exudes from the stem and branches of the mesquite, a mimosa, several species of which grow in Texas, New Mexico, and Arizona.—*St. Louis Med. Jour.*

---

UMBILICAL HERNIA.

Dr. Cleveland reported a case of umbilical hernia, occurring in a very fleshy woman, æt. 57. The case was reported on account of the apparent success of aspiration, which, in the hands of most surgeons, has been unsatisfactory. The strangulation had existed twenty-four hours before council was called in.

Vomiting continued in the meantime, and severe pain at the seat of the tumor.

The patient was kept under the influence of opium for about twenty-four hours, and ice constantly applied, without success; then he determined to aspirate.

The needle was introduced into the tumor, and fluid and gas escaped, causing the tumor to be softer. A number of punctures were made in the surrounding cellular tissues, allowing considerable serum to escape. Slight manipulation was again resorted to, but failed to relieve the strangulation; ice was reapplied.

In about one and a half hours the tumor disappeared, and the patient was entirely relieved. The ice was, of course, an important agent in the case, but the aspirator apparently facilitated the reduction.—*Clinic.*

---

INSTANTANEOUS CURE OF HYDROCELE.

Dr. Macario, of Nice, contributes to *L' Abeille Medicale* some interesting cases treated by electro-puncture. In the first case, two needles were plunged into the tumor, one at the base and the other at the apex. On connecting the needles the pain was such that the patient refused to continue treatment. Nevertheless, the next day the liquid had disappeared, and had not returned at the end of nine years. In the next case absorption was even more rapid, a tumor the size of two fists, dating from fifteen months, having vanished in the evening after a single sitting of one minute. Dr. M. has also reported to the Institute several other cases treated, some by electro-puncture, others by simple induced currents, and it is more than fifteen years since he first recommended this method, which has been followed by several others with considerable success.—*St. Louis Med. and Surg. Jour.*

---

TRACHEOTOMY.

Prof. Johnston, of the University of Maryland, in Baltimore Clinical Society, says of the method for performing this operation:

I would only remark that I prefer, and have always performed the operation taught me by Chassaignac, which is done by first securing the larynx by passing a tenaculum, grooved upon its convexity, under the cricoid cartilage, after the median cutaneous incision. Then, bearing the trachea, a pointed knife is made to penetrate the trachea by following the groove in the tenaculum-director; next a a blunt-pointed knife enlarges the tracheal opening, cutting the rings downwards; after this, Trosseau's ditator opens the slit; and the operation is completed by the introduction of a canula. Trosseau's double canula has had numerous rivals; but Durham's canula, which I now use almost exclusively, is, in my estimation, the very best which has yet been given to the profession.—*Maryland Med. Jour.*

---

Excessive lactation in nursing women is often one of the most difficult complications which we encounter in practice. It is not only unpleasant and painful, but frequently debilitating to the mother. An obstinate case of this character is mentioned by Rehan (*Przeglad lekarski*), in which frequent catharsis, with the internal administration of iron and quinia, failed to diminish the amount of milk. At the end of one month, during which the iodide of iron, quinia, and salicylic acid were given, and the induction current used, the amount of milk secreted was just one-third the previous quantity, and both mammæ were diminished in size. In two months, the woman had regained her former strength. The reporter of the *Allg. Med. Central Zeitung*, in commenting on this case, expresses the opinion that ergotin, either by the mouth or subcutaneously, would have produced the same results in a much shorter space of time.—*The Clinic, Jan.* 19, 1878.

---

GUTTA PERCHA TISSUE

Dr. Chamberlain gives an account, in the *New York Medical Record*, of a superior gutta percha tissue, which is rolled out as thin as fine French writing paper, being almost transparent, with a satiny luster. It is unaffected by the heat of the body, but softens at a somewhat higher heat. It is insoluble in water, but soluble in ether, chloroform, and alcohol. It is especially suited for wounds or lesions of the hands, forming a neat, light, clean, impervious dressing, allowing the hands to be put into water. Adhesion of cut surfaces and resolution of infiltrated deposits take place very quickly and kindly. If a broad patch of the skin is to be shielded from the air—*e. g.*, a scalded surface or a patch of eczema—a piece of the tissue somewhat larger is laid on the surface, and sealed in position by tracing the margin with a camel's-hair pencil dipped in chloroform, precisely as a covering-glass to a microscope-slide is adjusted.

---

COTA BARK IN DIARRHŒA AND RHEUMATISM.

This new bark, from Bolivia, is said, by Professor Giete, of Munich, to be a specific against diarrhœa in its most diverse forms. He administers it in doses of 0.5 gramme of the fine powder four or six times a day. Of the tincture, he usually gives ten minims every two hours. In Bolivia, whence the plant was sent, it is regarded as a remedy against rheumatism and gout.—*Virginia Med. Monthly.*

---

FOLICULAR STOMATITIS.

*Symptoms.* — Difficulty of sucking; abundant flow of saliva, sub-maxillary glands tumid and tender; restlessness and fever; loss of appetite; diarrhœa, with offensive motions; small vesicles on inside of mouth, on tongue and fauces. Vssicles burst and form ulcers, which are covered with dirty white or yellowish sloughs.

*Treatment.*—Application with a camel's hair pencil of borax and glycerine; mild tonics; carbonate of magnesia; chlorate of potash; attention to the milk supplied to the child; beef tea.—*Tanner.*

---

PHOSPHIDE OF ZINC IN HYSTERIA.

Dr. Em. Gros speaks of a case of long continued hysteria cured in five days by phosphide of zinc, from one to two-fifteenths of a grain in granules having been given with food thrice daily.

